# Presentations of Routine Dental Interventions in Children over a Six-Year Period

**DOI:** 10.1155/2022/9285893

**Published:** 2022-05-16

**Authors:** Samia Aboujaoude, Balsam El Noueiri

**Affiliations:** Department of Pediatric Dentistry, Faculty of Dental Medicine, Lebanese University, Beirut, Lebanon

## Abstract

**Aim:**

To evaluate over a six-year period, the prevalence of the dental procedures in primary and mixed dentitions, in males and females.

**Materials and Methods:**

A retrospective descriptive study on dental treatments in primary and mixed dentitions was conducted in the Department of Pediatric Dentistry at the Faculty of Dental Medicine, Lebanese University, from 2015 to 2020. The total number of children was 1291. Data were classified and reviewed according to the dentition type, gender, and type of dental treatment.

**Results:**

The number of children decreased from 741 for the period 2015–2017 to 550 during the period 2018–2020 (279 males and 271 females). No significant association was found between the admission periods and the genders. A significant relationship was found between the number of treatments performed in both genders and the dentition types (*p* value = 0,015). Males were slightly more likely than females to present for the treatment in mixed dentition (54.58% versus 45.2%, respectively). Results portrayed a significant relationship between surgical treatment and gender (*p* value = 0.049). However, no significant relationship between gender and other treatment types was noted. The comparison between the 2 time frames and the types of treatments showed a significant association in composite fillings (*p* value = 0.043), extractions (*p* value < 0.0001), sealants (*p* value = 0.039), preventive resin restoration (*p*=0.011), pulp therapies (*p* value < 0.0001), pediatric crowns (*p* value < 0.0001), and surgical interventions (*p* value = 0.013). A nonsignificant relationship was recorded for the appliances and composite crowns (*p* value = 0.45 and 0.14, respectively).

**Conclusion:**

The present study points out the implications of the COVID-19 outbreak and Lebanon's economic collapse on children's dental status, with the number of children receiving dental care dropping remarkably. A decrease in all types of dental procedures was noted in mixed dentition, whereas an increase in dental treatments related to aggravated carious lesions was reported in primary dentition. More medical and financial aids are required to encourage and support parents' attitude towards children dental care during unprecedented crises.

## 1. Introduction

Epidemiological studies are essential in oral health to diagnose current diseases and their treatment needs [1]. Similarly, they are crucial to draw the attention of policy makers in implementing required preventive actions [2, 3]. Dental caries is a major oral health problem affecting 35.3% of the global population [4].

According to the World Dental Federation (FDI), oral health plays a crucial role in achieving decent general health and enhancing quality of life [5, 6].

Dental caries commences at a very young age and is considered as a widespread oral disease in children around the globe [7].

In infants, early childhood caries (ECC) involves a distinctive pattern which severely degrades the primary maxillary incisors [8–10]. In mixed dentition, caries prevalence could be predictive of that in the permanent one. Therefore, early caries diagnosis is important to reverse the disease process, prevent progression of caries, and reduce incidence of future lesions [11–13]. Reports show that dental caries affects 60–90% of schoolchildren in both developing and developed countries [14, 15]. Some studies concluded that Middle Eastern schoolchildren are at a high-risk of developing dental caries with a prevalence up to 83.3% [16–18]. Moreover, recent investigations claimed that political and socioeconomic factors are directly related to parental healthcare accessibility, as well as their oral health knowledge and attitudes [19, 20].

Therefore, assessing the prevalence of conducted dental treatments in children becomes as important as assessing dental caries.

In Lebanon, COVID-19 pandemic struck at a time where the country was facing a major economic and political crisis, and as a result, many of the Lebanese population were incapable to sustain a proper oral healthcare [21].

The present study aims to assess conducted dental treatments in primary and mixed dentitions of young Lebanese patients between the periods 2015–2017 (precrisis period) and 2018–2020 (beginning of an economic and a pandemic crises) by

- Evaluating the prevalence of the dental procedures in primary and mixed dentitions
- Comparing all types of dental treatments between males and females

## 2. Materials and Methods

A retrospective descriptive study on dental treatments in primary and mixed dentitions was conducted in the Department of Pediatric Dentistry at the Faculty of Dental Medicine, Lebanese University, from 2015 to 2020. The total number of pediatric patients was 1291, 610 females and 681 males. All patients' records were collected from the DenTrooper logistic program, from the faculty archives, after research committee's approval (*n*#6941). Data were classified and reviewed according to two age groups (2–6 years old and 7–11 years old), gender (female or male), and type of treatments (composite restoration, pulp therapy, extraction, and pediatric crowns). Data collection and depiction are given in Tables 1–4.

### 2.1. Statistical Analysis

Data analysis was carried out using GraphPad Prism version 5 for Windows (GraphPad Software, La Jolla, California, USA, www.graphpad.com). Pearson's chi-square and Fischer's exact test were used to determine whether there is a statistical relationship between the nominal variables. *P*-value <0.05 was considered statistically significant.

The total number of children attending the Department of Pediatric Dentistry was 1291, among them 610 females (47.25%). The number of young patients was 741 children between 2015 and 2017. Among them, 45.7% were females and 54.25% were males. The number of children decreased from 741 for the period 2015–2017 to 550 during the period 2018–2020 (279 males and 271 females). Only eighty children were admitted in 2020. No significant association was found between the admission periods and the genders (*P* value = 0.21) (Table 1).

The number of dental treatments was 8777, among which 6929 (78.94%) were in mixed dentition with 45.2% females and 1848 (21.06%) were in primary dentition with 48.6% females. A significant relationship was found between the number of treatments performed in both genders and the dentition types (*P* value = 0.015) (Table 2).

The results showed the number of each type of dental treatments and its percentage, according to the gender. Results portrayed a significant relationship between surgical treatment and gender (*P* value = 0.049), while no significant relationship between gender and the other treatment types was found (Table 3).

For each type of dental treatment, the relationship between the types of dentition (primary or mixed) and the admission periods was tested. A significant association was found in composite fillings (*P* value = 0.043), extractions (*P* value < 0.0001), sealants (*P* value = 0.039), preventive resin restoration (*p*=0.011), pulp therapies (*P* value < 0.0001), pediatric crowns (*P* value < 0.0001), and surgical interventions (*P* value = 0.013). A nonsignificant relationship was noted for the appliances and composite crowns (*P* value = 0.45 and 0.14, respectively) (Table 4).

In mixed dentition, the number of composite restorations was 1261 (58.19%) for 2015–2017 and 906 (41.81%) for 2018–2020. The number of composite restorations in primary dentition was 357 (53.77%) in 2015–2017 and 307 (46.23%) in 2018–2020. The number (*n*) of extracted teeth in the mixed dentition stage was higher in 2015–2017(*n* = 470; 58.19%) than in 2018–2020 (*n* = 411; 41.81%). For the primary dentition, the number of composite restorations was higher in 2018–2020 (*n* = 127; 64.8%) than in 2015–2017 (*n* = 69; 35.2%).

## 3. Discussion

According to the World Health Organization reports, dental caries affects 60–90% of schoolchildren in both developing and developed countries [14, 15].

It is the most prevalent and costly disease among school-aged children around the world and imposes a financial burden on their families [19, 22, 23]. Socioeconomic factors are proven to have the possibility to influence the prevalence of dental caries in children [24].

In the present study, the sample of young patients attending the Department of Pediatric Dentistry at the Faculty of Dental Medicine, Lebanese University, have a low socioeconomic level background. Between the period 2015 and 2017, 741 children attended the Department of Pediatric Dentistry. The number decreased to 550 between 2018 and 2020 with only 80 children in 2020 (Table 1).

No statistical significance was noted between the two genders relative to the admission period (*P* value = 0.21).

The decline in the number of children (26%) between 2015–2017 and 2018–2020 could be related to the COVID-19 pandemic, aggravated by a severe socioeconomic crisis in the country. Several reports reveal that low income and increased poverty rates had a significant impact on overall oral health [25, 26].

Kharroubi [25] (2021) stated that COVID-19 pandemic had detrimental global repercussions on the economy. Moreover, in Lebanon, the pandemic overlapped with an economic crisis which overwhelmed the medical system [25]. In the same vein, Shallal et al. found that the financial crisis worsened, and the banking systems went completely bankrupt. With the dawn of the economic and political turmoil, the healthcare system continued to face difficulties due to shortage of supplies [26].

In Table 2, a significant relationship was found between the number of treatments performed in both genders and the dentition types (*P* value = 0,015). With respect to dentition type, males were slightly more likely than females to present for the treatment in mixed dentition (54.58% versus 45.2%, respectively).

The performed treatments in mixed dentition (6929) were remarkably higher than those performed in primary dentition (1848). These numbers can not only constitute sufficient evidence of parents' lack of awareness of their children's oral health and a neglect of seeking early dental care as stated by Setty and Srinivasan [27] but also could be attributed to the fact that children under 6 years old are directly and exclusively consigned to skilled residents in the master program. The residents are well trained to deliver care to very young children with limited cooperation due to anxiety, iatrophobia, or presenting special healthcare needs [28].

As the number of residents handling patients in primary dentition is very limited when compared to the number of undergraduate students who manage patients in mixed dentition, the difference between the number of performed treatments in primary and mixed dentition becomes understandable and comprehensible.

Data analysis related to dental treatment types and genders did not express a statistical significance except for surgical procedures where males were predominant (Table 3). Nevertheless, it cannot be taken into consideration as the sample size was limited.

In Table 4, the comparison between all types of dental treatment performed in 2015–2017 and the ones in 2018–2020 for both dentitions presented significant results in composite (*p*=0.043), extractions (*p*=0.0001), fissure sealants (*p*=0.039), PRR (*p*=0.011), pulp therapy (*p*=0.001), pediatric crowns (*p*=0.0001), and surgical procedures (*p*=0.013).

In primary dentition, between the 2 periods, the number of extractions, pulp treatments, and pediatric crowns increased from 69 to 127, 122 to 214, and 195 to 247, respectively, while the number of appliances, composite restorations, and fissure sealants decreased. It might be due, on the one hand, to the country's economic collapse which generally affected families' financial situations and led them to reconsider prioritizing their expenses. On the other hand, it could be related to the pandemic's confinement and restriction measures [29]. Both crises could be the major cause of the delay in seeking dental care and led to aggravated dental lesions [29]. An international survey conducted in 2021 demonstrated that dental practice in several countries was limited to emergencies [30].

In mixed dentition, a decrease in all types of dental treatments was observed between the 2 periods of time (2015–2017 and 2018–2020). The reduction in the number of procedures could be a result of the COVID-19 confinement and the devaluation of the Lebanese currency which created a severe socioeconomic burden on the Lebanese population. In 2021, Shallal et al. stated that the Lebanese pound lost 80% of its value and the poverty rate increased from 28% in 2019 to 55% in 2020 [26]. She also declared that the COVID-19 outbreak has affected individual behavior, lives, and professions worldwide [26]. Similarly, Koweyes et al. (2021) reported that one year of the pandemic rushed the collapse of the ongoing economic, political, and health sectors, in Lebanon [31].

## 4. Conclusion

The present study highlights the implications of both the COVID-19 outbreak and Lebanon's economic collapse on children's dental status, with the number of children receiving dental care dropping remarkably. Moreover, a decrease in all types of dental procedures was noted in mixed dentition, whereas an increase in dental treatments related to aggravated carious lesions, such as extractions, pulp therapy, and pediatric crowns, was reported in primary dentition.

Therefore, dental awareness programs must be designed to improve children's oral health care and parents' behavior. Nevertheless, more medical and financial aid is required to encourage and support parents' attitude towards children' dental care during unprecedented crises.

## Figures and Tables

**Table 1:** Comparison between the admission periods and the genders.

	Female		Male		Total	*P* value
Period	Nb	%	Nb	%	Nb
2015–2017	339	45.75	402	54.25	741	0.21
2018–2020	271	49.27	279	50.73	550	
Total	610	47.25	681	52.75	1291	

**Table 2:** Comparison between the number of dental treatments performed in both genders and the dentition types.

	Female		Male		Total	*P* value
Dentition	Nb	%	Nb	%	Nb
Primary	898	48.6	950	45.42	1848	0.015
Mixed	3147	45.2	3782	54.58	6929	
Total	4045	46.09	4732	53.91	8777	

**Table 3:** Comparison between each type of dental treatment and gender types.

All type of dental treatments	Female		Male		Total	*P* value
	*N*	%	*N*	%		
Appliance	162	46.02	190	53.98	352	0.95
Composite	1309	46.24	1522	53.76	2831	0.88
Restoration with celluloid mold	34	58.62	24	41.38	58	0.0547
Extraction	488	45.31	589	54.69	1077	0.058
Fissure sealant	525	45.45	630	54.55	1155	0.834
Preventive resin restoration (PRR)	223	45.70	265	54.30	488	0.85
Pulp therapy	587	47.92	638	52.08	1225	0.165
Pediatric crown	713	45.36	859	54.64	1572	0.52
Surgical procedure	4	21.05	15	78.95	19	**0.049**
Total	4045	46.09	4732	53.91	8777	

**Table 4:** Comparison between all types of dental treatments performed in 2015–2017 and those performed between 2018 and 2020 in both dentitions.

Type of dental treatments	Period	MD		Primary		Total	*P* value
		*N*	%	*N*	%		
Appliance	2015–2017	211	66.56	21	60.00	232	0.45
	2018–2020	106	33.44	14	40.00	120	
Composite	2015–2017	1261	58.19	357	53.77	1618	**0.043**
	2018–2020	906	41.81	307	46.23	1213	
Restoration with celluloid mold	2015–2017	11	58.19	18	43.90	29	0.14
	2018–2020	6	41.81	23	56.10	29	
Extraction	2015–2017	470	58.19	69	35.20	539	**0.0001**
	2018–2020	411	41.81	127	64.80	538	
Fissure sealant	2015–2017	590	58.19	38	67.86	628	**0.039**
	2018–2020	509	41.81	18	32.14	527	
Preventive resin restoration (PRR)	2015–2017	263	58.19	29	45.31	292	**0.011**
	2018–2020	161	41.81	35	54.69	196	
Pulp therapy	2015–2017	527	58.19	122	36.31	649	**0.0001**
	2018–2020	362	41.81	214	63.69	576	
Pediatric crown	2015–2017	689	58.19	195	44.12	884	**0.0001**
	2018–2020	441	41.81	247	55.88	688	
Surgical procedure	2015–2017	1	58.19	14	100.00	15	**0.013**
	2018–2020	4	41.81	0	0.00	4	
Total		6929		1848		8777	

## Data Availability

The data used to support this study are included within the article.
